# Lectures on the Operations of Surgery, and on Diseases and Accidents Requiring Operations

**Published:** 1846-08

**Authors:** 


### ARTICLE XIII.

*Lectures on the Operations of Surgery, and on diseases and accidents requiring Operations.* By Robert Liston, Esq., F. R. S., Senior Surgeon to the University College Hospital, &c. &c., with numerous additions by Thos. D. Mütter, M. D., Prof. of Surgery in Jefferson Medical College, Philadelphia, etc. etc. etc. Philadelphia: Lea & Blanchard. 1846. pp. 565. 8 vo. (From the Publishers.)

This work consists of a series of lectures published in the London Lancet, and now collected in a volume. The works of Mr. Liston are all characterized by a vigor of style and energy of thought, which taken in connexion with his boldness as a surgeon, renders them very interesting. The present work is not an exception. Scarcely any operation is declined by Mr. Liston, on account of its difficulty or severity, and this is also true of the editor. They both join, however, in condemning the extirpation of diseased ovaria, but the data and reasons upon which these opinions rest, are, perhaps, not sufficient to set the question at rest in the minds of the profession at large.

The additions of Prof. Mütter equal in quantity the lectures themselves, and consist in a great measure of papers which have already been laid before the public, through the medium of medical journals. It were needless to say that this work contains much valuable matter; yet it is with regret that we see the medical literature of our country, assuming so entirely, as it has done of late, the form of articles for periodicals and notes to foreign works, and we hope that Prof. Mütter is not in this manner to disappoint us of his promised system of Surgery.

